# Validation of reference genes for quantitative PCR in the forest pest, *Ips calligraphus*

**DOI:** 10.1038/s41598-021-02890-z

**Published:** 2021-12-07

**Authors:** Mary Wallace, Lynne K. Rieske

**Affiliations:** Department of Entomology, University of Kentucky, Lexington, KY 40546 USA

**Keywords:** Biological techniques, Molecular biology

## Abstract

The six-spined ips, *Ips calligraphus*, is a North American bark beetle that can exploit most eastern North American *Pinus* species and can cause mortality. Biotic and abiotic disturbances weaken trees, creating breeding substrate that promotes rapid population growth. Management historically relied on silvicultural practices, but as forests become increasingly stressed, innovative management is needed. Manipulation of the cellular RNA interference (RNAi) pathway to induce gene silencing is an emerging means of insect suppression, and is effective for some bark beetles. Quantitative PCR (qPCR) is a powerful tool for analysis of gene expression, and is essential for examining RNAi. To compare gene expression among individuals, stably expressed reference genes must be validated for qPCR*.* We evaluated six candidate reference genes (*18s*, *16s*, *28s*, *ef1a*, *cad*, *coi*) for stability under biotic (beetle sex, developmental stage, and host plant), and abiotic (temperature, photoperiod, and dsRNA exposure) conditions. We used the comprehensive RefFinder tool to compare stability rankings across four algorithms. These algorithms identified *18s*, *16s*, and *28s* as the most stably expressed. Overall, *16s* and *28s* were selected as reference genes due to their stability and moderate expression levels, and can be used for *I. calligraphus* gene expression studies using qPCR, including those evaluating RNAi.

## Introduction

**Figure 1:**
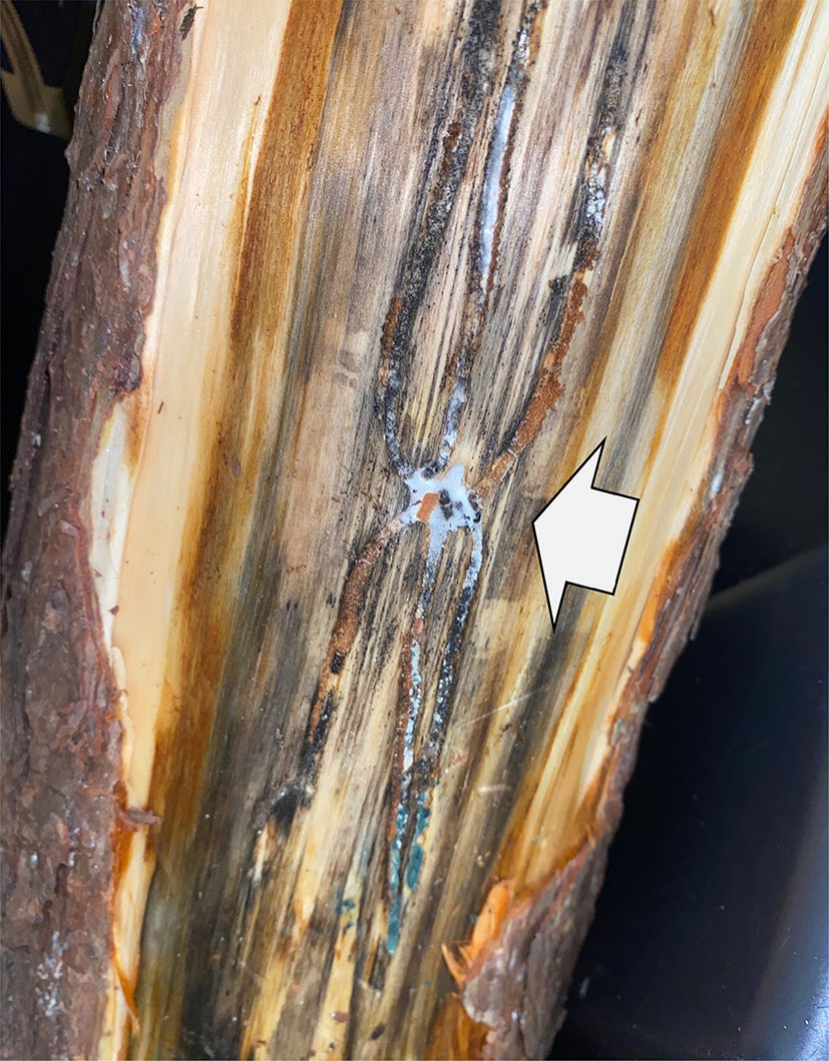
*I. calligraphus* gallery in a debarked *P. taeda* log showing an adult beetle with fungal growth, and blue staining associated with *Ophiostoma* spp.

*Ips calligraphus*, the six-spined ips, is a native North American bark beetle that can infest most *Pinus* species, including economically important species such as loblolly (*P. taeda*), shortleaf (*P. echinata*), and slash (*P. elliottii*)^1–3^. Historically, *I. calligraphus* has been considered a secondary pest, utilizing volatile compounds produced by stressed trees to preferentially locate and infest those already in poor condition, then using sex pheromones to further attract conspecifics to suitable hosts^4^. Adults mate, and females then excavate oviposition galleries (Fig. 1), where eggs are laid and larvae develop, feeding within the phloem^3^. In the southeast region of the United States development requires 15–40 days (mean 25 days), allowing for multiple generations per year^2^. Typically, *I. calligraphus* infested trees are spread sporadically throughout the forest, and no management is necessary^5^. However, the increasing frequency and intensity of regional abiotic extremes^6–8^ has generated disturbed sites with an abundance of suitable breeding material, creating the potential for rapid population growth^9,10^. These post-disturbance population increases have elevated *I. calligraphus* beyond its status as a secondary pest to one responsible for significant economic losses^5,11,12^. Furthermore, *I. calligraphus* has historically been invasive in the Philippines and Jamaica^5,13^, and has more recently established populations in south mainland China^14^, well outside of its native range, and is classified as a Scolytinae at high risk of becoming invasive in the southern hemisphere^15^. This invasiveness, coupled with human-mediated disturbances and rapidly changing climatic conditions, has increased the risk that *I. calligraphus* poses to forest ecosystems, necessitating development of innovative, sustainable, and affordable management tools.

Management techniques relying on the knowledge and manipulation of genetic information have recently been deployed against agricultural pests^16,17^, but are only just emerging as a potential means of reducing forest pest populations^18–21^. Manipulating the RNA interference (RNAi) pathway serves as one example of this technology, exploiting the insect’s own viral defense mechanism^22^ to target critical mRNAs, thus generating mortality in the target insect while showing extreme specificity, minimizing concerns of non-target effects^21,23,24^. RNAi functions through the introduction of species-specific double-stranded RNA (dsRNA), which is recognized by the endogenous RNAi pathway present in the target insect. The dsRNA is then cleaved by the enzyme dicer into 21–23 base pair strands known as small-interfering RNA (siRNA), which are incorporated into the RNAi-induced silencing complex (RISC)^25^. The siRNA is then used as a template for the RISC complex to bind to and cleave complementary mRNA using an Argonaute enzyme, preventing translation into protein^25^. If the targeted genes are essential, this process can cause insect mortality. Considering there must be a precise ≥ 16 base pair match, or > 26 base pair near-perfect match for the degradation to occur^24^, RNAi can be highly specific to both the species and transcripts being targeted, minimizing the risk of non-target effects, an important consideration for deployment. Given the susceptibility of other scolytines to RNAi, including the southern and mountain pine beetles, *Dendroctonus frontalis*^19^ and *D. ponderosae*^20^, the potential for developing and utilizing RNAi as a tool for *I. calligraphus* management is promising.

An established method of demonstrating gene silencing through the introduction of exogenous dsRNA involves evaluation of relative gene expression via quantitative PCR (qPCR). This requires selection of stably expressed endogenous controls, or reference genes, that can be used to interpret gene expression levels across samples that may have different mRNA levels due to methodological variation, rather than biological causes^26,27^. Commonly selected reference genes are those that are critical to cellular function, as these genes are most often stably expressed across cell types and environmental conditions. Reference genes must be validated across conditions relevant to a given experimental design before use as an endogenous control for qPCR in gene expression studies. The stability of a candidate reference gene can be quantitatively evaluated via algorithms such as GeNorm, NormFinder, delta-Ct, and BestKeeper, allowing for selection of the most reliable endogenous controls.

The goal of the present study is to identify stably expressed reference genes for use in relative gene expression analysis in *I. calligraphus*, including those valid for studies of exogenous dsRNA induced gene silencing. We evaluated six candidate housekeeping genes for stability across several biotic (beetle sex, developmental stage, and host plant) and abiotic (temperature, photoperiod, dsRNA exposure) conditions. This is the first validation of reference genes for *I. calligraphus*, and is a necessary step for future qPCR gene expression analyses. qPCR is essential for demonstrating gene knockdown from dsRNA exposure, which is the next step in working toward developing an RNAi-based pest suppression strategy for *I. calligraphus*.

## Results

**Figure 2:**
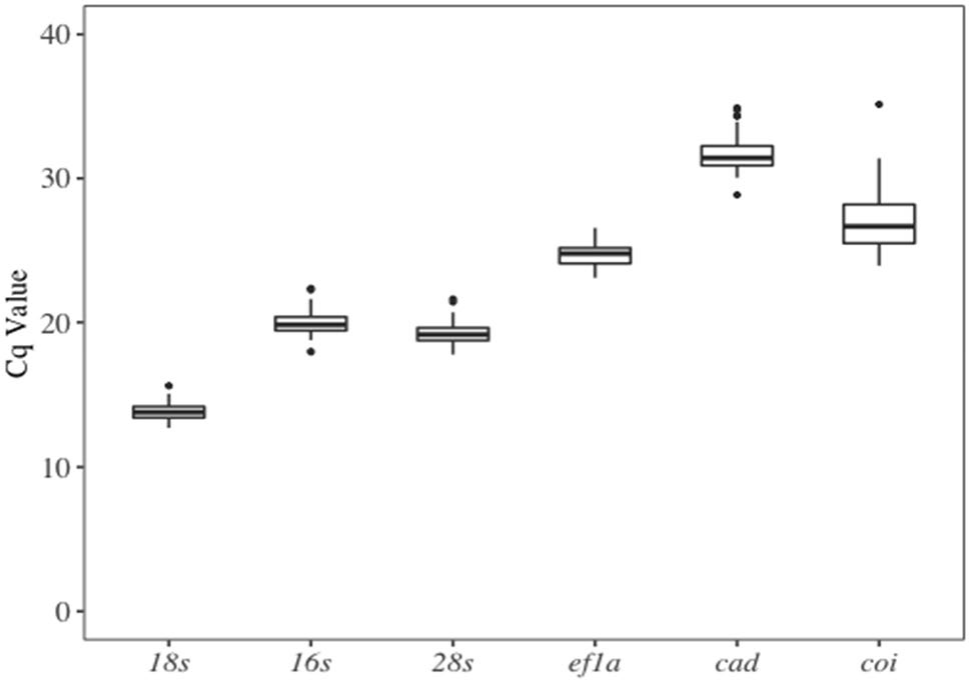
Cq values for each candidate reference gene across all treatments. Boxes encompass the 25th to 75th percentiles, and whiskers represent 1.5 times the interquartile range. Outliers are indicated by dots.

Across all treatments combined (Fig. 2) the most highly expressed gene is 18s ribosomal RNA (*18s*) with Cq values ranging from 12.7 to 15.6, while Ca2 carbamoyl-phosphate synthetase 2 (*cad*) has the lowest expression with Cq values ranging from 28.8 to 34.9.

Cytochrome oxidase I (*coi*), has the greatest range of expression, with Cq values ranging from 23.9 to 35.1. Of the remaining genes evaluated, 16s ribosomal RNA (*16s*), 28s ribosomal RNA (*28s*), and elongation factor 1-alpha (*ef1a*), are moderately expressed, with average Cq values ranging from 19.2 to 24.7.

**Figure 3:**
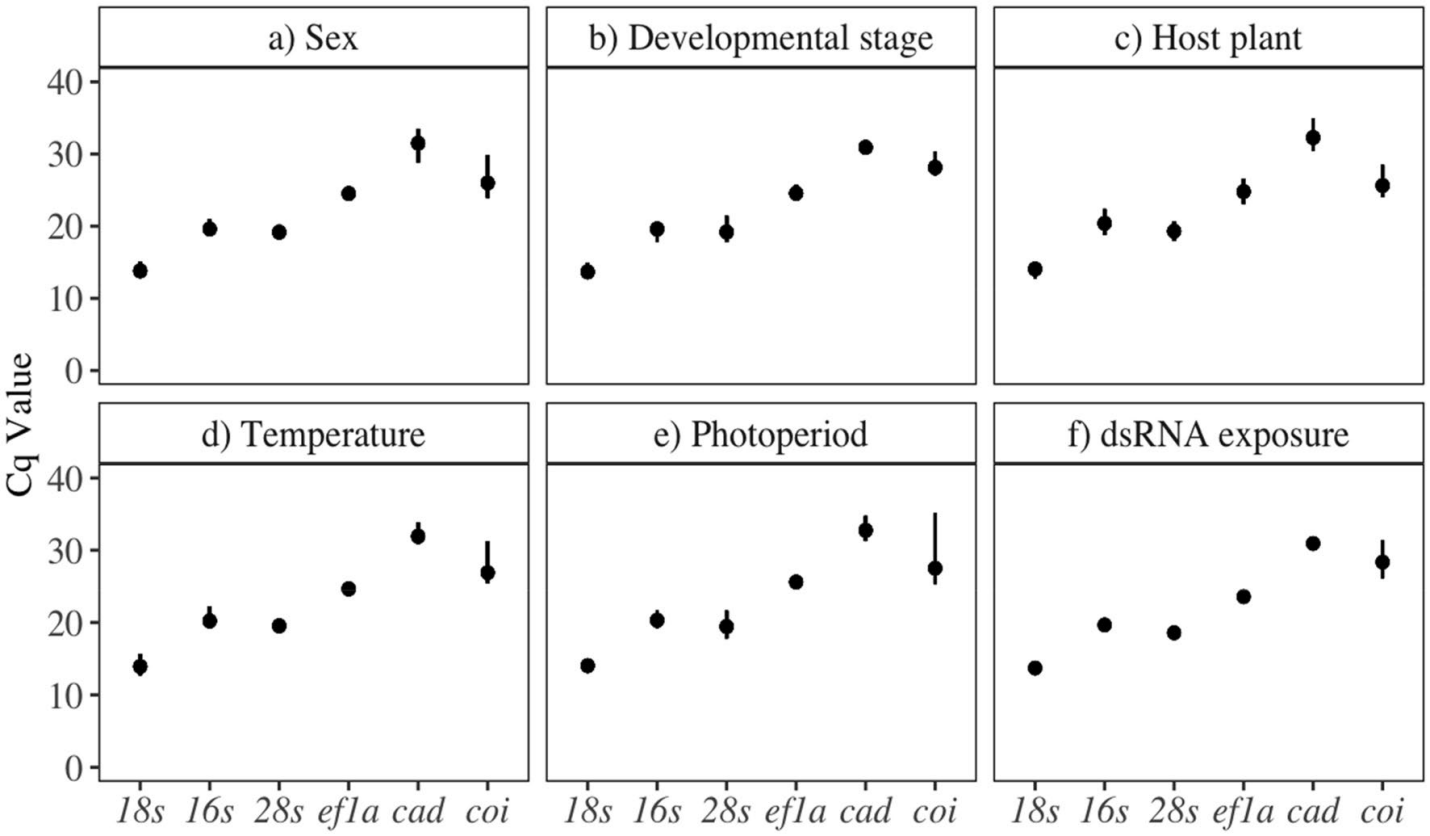
Average Cq values for each candidate reference gene by: (**a**) beetle sex, (**b**) developmental stage, (**c**) host plant, (**d**) temperature, (**e**) photoperiod, and (**f**) dsRNA exposure. Error bars represent the maximum and minimum Cq values, n = 5 beetles per treatment.

The average Cq value of each candidate reference gene was determined for each condition, including beetle sex, developmental stage, host plant, temperature, light conditions, and dsRNA exposure (Fig. 3a–f). The relative level of expression for each candidate gene was consistent across all conditions, with *18s* having the highest expression, and *cad* being the least expressed. Across all conditions tested, *coi* was the most variable, having the largest range of Cq values.

**Figure 4:**
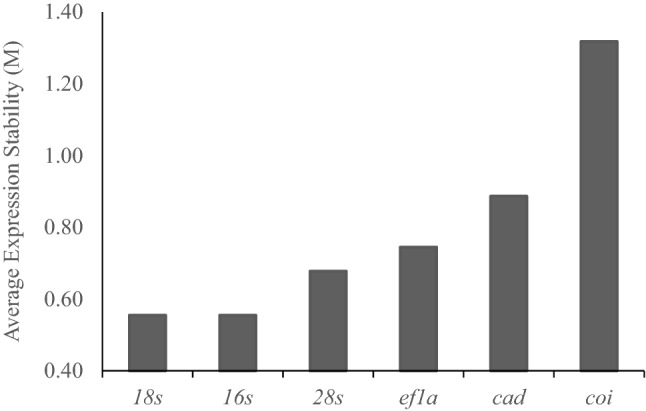
Average expression stability values (M) of the 6 candidate reference genes plotted from most stable (left) to least stable (right) analyzed by the GeNorm software.

GeNorm^28^ uses a gene expression stability value (M) to determine stability; M < 1.5 is required for a gene to be considered stable. All candidate genes tested had an M value below 1.5, with *16s* and *18s* being ranked equally as most stable (Fig. 4). NormFinder^28^ ranks candidate genes based on an overall stability value (SV); SV > 1 indicates lower stability. *18s* and *16s* had the lowest SV scores, with *28s* and *ef1a* being the only other candidate genes to meet the threshold. BestKeeper^28^ uses standard deviation (SD) to determine stability, with a SD < 1 more stable. Using this approach, all candidate genes except *coi* met the threshold for stable expression, but *18s*, *28s*, and *16s* were ranked as the most stable. The comparative delta-Ct method also uses SD to determine stability, with a lower value indicating a more stable expression. Using the delta-Ct method, *18s* and *16s* were the most stable.

The geomean value was determined using the web tool RefFinder^28^, which gave a comprehensive ranking across the four algorithms, with *18s* ranking first, *16s* ranking second, and *28s* ranking third. The four top-ranking reference gene candidates met the stability parameters of each of the algorithms (Table 1). Of these, *16s* and *28s* were selected as reference genes, as they have similar levels of stability to *18s* across the algorithms used, while having a more moderate Cq values, allowing them to be more applicable to evaluating genes with moderate levels of expression.

**Table 1:** Stability rankings of the 6 candidate reference genes using RefFinder: GeNorm, NormFinder, BestKeeper, delta-CT, and a comprehensive rank from RefFinder.

Gene	GeNorm		NormFinder		BestKeeper		delta-Ct		Comprehensive	
	M	R	SV	R	SD	R	SD	R	GM	R
*18s*	0.56	1	0.17	1	0.50	1	1.01	1	1.00	1
*16s*	0.56	1	0.46	2	0.64	3	1.08	2	1.86	2
*28s*	0.68	2	0.73	4	0.59	2	1.18	4	3.13	3
*ef1a*	0.75	3	0.52	3	0.65	4	1.10	3	3.46	4
*cad*	0.89	4	1.02	5	0.91	5	1.36	5	5.00	5
*coi*	1.32	5	2.09	6	1.64	6	2.18	6	6.00	6

R: ranking; M: gene expression stability; SV: stability value; SD: standard deviation; GM: geomean value.

## Discussion

qPCR is an indispensable tool for evaluating gene expression, an important aspect of many genetic studies, and essential for demonstrating RNAi-induced gene silencing, as its sensitivity permits detection of even minor differences between samples^27,29,30^. However, this sensitivity can also be a limitation, as any differences in baseline transcription levels, variation in sample preparation, or technical inconsistencies become evident. To normalize gene expression between samples for qPCR quantification of mRNA levels, internal controls that are stably expressed in various experimental conditions are needed^26^. The selection and use of stable reference genes allow researchers to normalize variation between samples, thus giving a more accurate representation of gene expression differences and avoiding error.

Despite some variation in ranking, the top four reference genes (*18s*, *16s*, *28s*, and *ef1a*) were consistently stable regardless of the algorithm used. In addition, they were consistently stable across the biotic and abiotic conditions we tested, and these conditions may vary across different experimental designs, increasing the versatility of our selected reference genes for use in gene expression studies. The conserved nature of these essential genes should also allow for their use in genetically variable individuals, though further investigation is required to confirm this among different populations or subspecies. Stability in dsRNA-exposed beetles and individuals of different developmental stages could allow for these same reference genes to be used for normalizing gene expression in RNAi experiments using oral delivery of dsRNA in a sucrose solution, with treatment at multiple life stages, an important factor given that *I. calligraphus* causes damage both through larval feeding, and during dispersal as adults by carrying damaging microorganisms to naïve hosts. Additionally, RNAi manipulations allow for investigations into gene function using reverse genetics, serving as a technique that can illuminate the effects of reduced gene expression at a variety of life stages, including genes for which knockouts prevent complete development.

This study provides an essential foundation for future gene expression work in the experimental conditions analyzed. Additionally, the selection of reference genes that are stable for dsRNA treated beetles will serve as an important guide for their selection in future RNAi studies, a valuable tool of reverse genetics, and importantly a developing management tool, though additional efforts to verify reference gene stability across the specific conditions used in future RNAi studies are required. Due to the requirement of a ≥ 16 base pair match for gene silencing to occur^24^, with careful selection of the target gene sequences dsRNAs can be engineered to be highly specific to the target insect. Thus, utilizing RNAi for pest suppression could offer a means for managing for *I. calligraphus* while minimizing harmful effects on non-target organisms, one barrier to use of traditional insecticides to prevent and reduce outbreak populations. Further work establishing the practicality of potential methods of delivering dsRNA, such as root drenches, trunk injections, or transgenically expressed dsRNA, will be critical to deployment of this innovative technology, and will enhance our understanding of how it may be implemented in a forest ecosystem.

Climate change, with associated shifts in temperature and rainfall, have made forests more susceptible to large outbreaks in which stressed, and even healthy trees are killed. In these conditions, traditional management techniques like silvicultural control measures are not sufficient to promote healthy forests, and begin to lose their efficacy. These concerns, in addition to the threat that the beetle may pose in its expanding introduced ranges, mean that a new technique must be added to our existing integrated pest management strategies, for which RNAi could be invaluable. Moreover, *I. calligraphus* represents just one species in a genus comprised of multiple devastating forest pests, including the massively destructive *Ips typographus*. Outbreaks of *I. typographus* have caused unparalleled damage in European forests, killing millions of trees and even nearly eliminating its primary host, Norway spruce (*Picea abies*), in affected areas^31^. These outbreaks are devastating ecologically, representing the loss of key carbon sinks and wildlife habitat, as well as a loss of genetic diversity associated with old growth forests that will be vital to adaptation and survival of these forests in the face of rapidly changing environmental conditions^31^. *I. typographus* has been intercepted repeatedly at North American ports^32^, and when coupled with its potential for expanding suitable ranges with climate change^33,34^, it becomes evident that forests across the globe are at great risk of damage by this beetle. As *I. calligraphus* outbreak populations respond to increasingly frequent disturbance events and invade naïve ranges, and other *Ips* species continue to devastate forests globally, the ability to apply modern molecular techniques in innovating new management approaches is critical.

## Materials and methods

### Gene selection

Candidate reference genes (Table 2) were selected because they are transcribed in all cells and have essential functions that are ubiquitously expressed across cell types. Sequences were obtained from previous entries to NCBI, and primers were designed using the online tool Primer3Plus^35^. Primer pairs with an amplicon length between 80–120 bp, a GC% of ~ 50%, and a melt temperature of 60 °C, as well as with the lowest self and any scores, were selected. Primers were only selected if the linear regression coefficient (*R*^2^) was > 0.99 and efficiency percentage was 90–110%.

**Table 2:** Six candidate reference genes, with their qPCR primer sequence, R^2^ correlation coefficient, percent primer efficiency, and accession number.

Gene name	Sequence 5′3'	R^2^	%E	Accession number
*18s rRNA*—18S Ribosomal RNA	CACCGGAAGGATTGACAGAT	0.99	95.8	KJ531060.1
	GTGGAGCGATTTGTCTGGTT			
*16s rRNA*—16S Ribosomal RNA	CAAACCTTTCATTCCAGCTTTC	0.99	103.8	AF397475.1
	AAAATACTGCGGCCGTTAAA			
*28s rRNA*—28S Ribosomal RNA	TCGACCTCTGGTGACTGTTG	0.99	105.4	KJ531116.1
	ACTTTCAGGACCCGTCTTGA			
*ef1a*—Elongation Factor—1 alpha	TTGGAACCATCCACCAAGAT	0.99	91.9	KJ531172.1
	GATGCTTTGGATGCCATTCT			
*cad*—Carbamoyl-Phosphate Synthetase 2	CGACATTTTGGGGTTGTAGG	0.99	90.0	KF862878.2
	ATTGAAGTGAACGCCAGGTT			
*coi*—Cytochrome Oxidase I	CATGGGGCTCAAATTTCCT	0.99	95.9	AF113335.1
	CACAGGAGTCATTCTTGCCA			

### Insects

Experimental insects (*Ips calligraphus calligraphus*) were obtained by suspending *I. calligraphus* lures (Synergy Semiochemicals Corporation, Delta, BC) on a plantation located on Florida Forestry Service land in Newnans Lake State Forest in central Florida for a two-week period in January 2020. Infested trees were felled by Florida Forestry Service personnel in compliance with institutional, national, and international guidelines addressing collections of plant material, and in compliance with IUCN provisions. Stems were immediately transported to the University of Kentucky, Lexington, KY, and stored at 4 °C. Stems were sectioned and transferred to rearing bins (55.6 × 62.7 × 81.3 cm) as needed. Beetles used to evaluate stability of gene expression based on sex and developmental stage were processed immediately, otherwise newly emerged beetles were maintained under specified conditions for 72 h to assess the stability of potential reference genes^18,19^. Beetles were sexed using the presence or absence of the pars stridens and the number of protibial spurs^36^. To evaluate developmental stage, adults, pupae, and larvae were collected by debarking an infested log and preserving for RNA extraction immediately. To evaluate reference stability of gene expression based on host plant, beetles were starved for 24 h, then fed white (*P. strobi*) or loblolly pine bark; beetles for each treatment fed on the same piece of bark, and feeding was confirmed by visual inspection of beetles within galleries and the presence of frass. Beetles were maintained at 20 °C and 25 °C to evaluate stability of gene expression based on temperature, and 16:8 L:D and total darkness to evaluate stability based on photoperiod. dsRNA treated beetles were individually fed 10 μg of green fluorescent protein dsRNA, and then after their four-hour feeding period kept for 72 h before RNA preservation. Excluding the sex and developmental stage treatments, all beetles were maintained in each respective condition for 72 h before being crushed directly into TRIzol reagent (Life Technologies, Carlsbad, CA) to ensure maximum RNA yield.

### Gene expression

Total RNA was extracted using TRIzol reagent, precipitated using isopropanol, and washed twice with 75% ethanol before being resuspended in nuclease free water. Nanodrop spectrometry was used to ensure RNA integrity, followed by cDNA synthesis with 400 ng total RNA, using the SuperScript III Reverse Transcriptase protocol according to manufacturer’s protocols. cDNA synthesis was followed by quantitative PCR on a fivefold dilution of cDNA, starting at a 1:25 dilution with each primer pair to calculate percent primer efficiency, to check for single melt curve to ensure no primer dimers, and to determine the ideal concentration to evaluate treatments (three technical replicates each). Quantitative PCR gene expression analysis was performed on cDNA of each individual beetle (diluted 1.6 E-3) (n = 5) from each treatment with the six primer pairs. Expression was determined for each candidate gene using the Cq (quantification cycle) value, the number of PCR cycles required to meet a detection threshold. All qPCR reactions were performed with the QuantStudio 3 Real Time PCR System (ThermoFisher Scientific, Waltham, MA) using PowerUp SYBR Green Master Mix (ThermoFisher Scientific, Waltham, MA). PCR reactions were performed (at 50 ℃ for 2 min, 95 ℃ for 2 min, (95 ℃ 1 s, 60 ℃ 30 s) × 40), followed by a melt curve analysis (at 60 ℃ 1 min, 95 ℃ 15 s), and each plate included a no template control for each primer pair.

### Stability analysis

Using the mean Cq value for each beetle per primer pair, stability was analyzed using RefFinder, which combines GeNorm, NormFinder, BestKeeper, and the delta-cq method, and produces a final ranking of the potential reference genes based on the geometric mean of the four methods^28^.

### dsRNA synthesis

Traditional PCR was performed using OneTaq 2X Master Mix with Standard Buffer (New England Biolabs, Ipswich, MA) from purified PCR template (Table 3) (at 94 ℃ for 30 s, (94 ℃ 30 s, 60 ℃ 1 min, 68 ℃ 1 min) × 30, 68 ℃ 5 min, 4.0 ℃ ∞), and PCR products were purified using the QIAquick PCR purification kit (Qiagen, Germantown, MD). This purified PCR product was then used to synthesize dsRNA using the MEGAscript RNAi Kit (Invitrogen, Waltham, MD). The quality and quantity of dsRNA was tested using NanoDrop spectrometry and gel electrophoresis.

**Table 3:** Green fluorescent protein dsRNA forward and reverse primers, with T7 promoter sequence.

Gene name	Sequence 5′3'
*dsGFP*—green fluorescent protein	TAATACGACTCACTATAGGGCGATGCCACCTACGGCAA
	TAATACGACTCACTATAGGGTGTCGCCCTCGAACTTCA

### Administering dsRNA

To administer the dsRNA treatments, 10 μg of dsRNA was suspended in a 0.5% sucrose solution containing 0.5% food dye in a 0.5 mL microcentrifuge tube, for a total of 4 μL of solution. The anterior end of each individual beetle was immersed in the solution up to the pronotum, so as to not interfere with spiracles and oxygen uptake. A KimWipe (Kimtech, Neenah, WI) was placed into the tube to prevent beetles from backing out until the solution was completely consumed (~ 4 h)^20^. After treatment exposure, beetles were placed in petri dishes (60 × 15 mm) lined with moistened KimWipes, and oriented vertically in a cylindrical humidity chamber (~ 23 × 21 cm) for 72 h at 23℃.
